# Key directions of fundamental research on bacteriophage Receptor Binding Proteins with applied potential

**DOI:** 10.3389/fmicb.2026.1834523

**Published:** 2026-07-21

**Authors:** Bożena Szermer-Olearnik, Tomasz Mrokowski, Andrzej Gamian, Karolina Filik-Matyjaszczyk

**Affiliations:** 1 Laboratory of Biomedical Chemistry, Department of Experimental Oncology, Hirszfeld Institute of Immunology and Experimental Therapy PAS, Polish Academy of Science, Wroclaw, Poland; 2 Laboratory of Medical Microbiology, Department of Immunology of Infectious Diseases, Hirszfeld Institute of Immunology and Experimental Therapy PAS, Polish Academy of Science, Wroclaw, Poland

**Keywords:** bacteriophages, host-range, pathogen detection, Receptor Binding Proteins, tail fibers, tail spikes, tailocins

## Abstract

Over the past decade, interest in research on bacteriophage Receptor Binding Proteins (RBPs) of tailed bacteriophages has increased substantially, reflecting a broader resurgence in phage research. Once underestimated, RBPs have now become the focus of extensive investigation, largely due to their promising applications in pathogen diagnostics, host-range engineering for improved phage therapy, and the development of tailocins, protein-based antibacterial agents capable of inhibiting or eliminating bacterial cells. This review aims to synthesize current knowledge on RBPs function and applications, with particular emphasis on their translational potential. We systematically analyzed recent literature, focusing on structural, biochemical, and functional studies that elucidate mechanisms of host recognition and explore emerging biotechnological and therapeutic uses. The reviewed studies highlight significant advances in understanding RBPs specificity, modular architecture, and adaptability. These features enable targeted bacterial recognition and manipulation of host range. Furthermore, we demonstrate that RBPs can be effectively engineered to be repurposed for diagnostic and antimicrobial applications. Together, these findings position RBPs as a foundational platform for next-generation phage-based technologies and their translation into clinical and industrial applications.

## Introduction

The tail is a multiprotein complex present in most bacteriophages and plays a central role in host recognition and genome delivery. The tail structure enables the injection of genetic material into the bacterial cytoplasm without disrupting cellular integrity. Historically, three main morphotypes have been distinguished among tailed bacteriophages. Phages with short, non-contractile tails classified as *Podoviridae*. Phages with long, non-contractile tails—*Siphoviridae*. As well as phages with long, contractile tails belonging to *Myoviridae*. Currently, according to the latest classification by the ICTV, the morphology-based families and the order *Caudovirales* were abolished and replaced by the class *Caudoviricetes* which groups all tailed bacteriophages with icosahedral capsids and double-stranded DNA (Turner et al., 2023). Although tail architecture adapts to specific host groups, two fundamental structural features are conserved. First, a central tubular structure composed of Tail Tubular Proteins (TTP) forms a channel for DNA ejection. Second, is the tube surrounded by tail fibers (TFP) or tail spikes (TS), which mediate the initial stages of bacterial host recognition and are collectively referred to as Receptor Binding Proteins (RBPs) (Cuervo et al., 2013). Adsorption and genome ejection represents essential steps in the bacteriophage life cycle. Consequently, RBPs—responsible for the specific host recognition—constitute key determinants of phage infectivity and evolutionary success (Hu et al., 2013).

The attachment of a bacteriophage to its host cell is a complex process that involves conformational changes resulting in irreversible binding. It mainly consists of a combination of biophysical changes taking place on the host surface, and there is a direct mechanistic relationship between RBPs and the phage adsorption process. Adsorption begins when bacteriophage recognized and binds to a suitable receptor, leading to irreversible adhesion. This step is not only crucial for successful phage infection and evolutionary fitness, but is also one of the factors determining phage specificity toward particular bacterial strains. Key factor underlying that success is the functionality of RBPs, as subsequent infection cannot occur without highly specific receptor binding. Another important aspect of that process is the kinetics of the adsorption process, which could be influenced by numerous variables such as host condition or physicochemical properties of the surrounding medium. Together, these factors determine the efficiency of phage attachment and, consequently, the host's susceptibility (Storms and Sauvageau, 2015). A detailed explanation of phage adsorption, focusing on the specific interactions between virus particles and the bacterial cell surface, was proposed by Anderson in 1949. He highlighted three key observations according to phage-host interaction: (1) phage-bacterium interactions are highly specific, (2) under undisturbed conditions, nearly every collision results in irreversible attachment, and (3) under vigorous agitation, almost no collisions lead to stable attachment. The initial bond to the host bacteria is sufficiently stable but can be disrupted by strong agitation, which in fact can prevent irreversible attachment. Tail fibers (RBPs) play a crucial role in preventing diffusion away from the cell surface prior to receptor recognition (Anderson, 1949; Storms and Sauvageau, 2015). For example, in phages T4 (Goldberg et al., 1994) and T7 (Hu et al., 2013), tail fibers have adapted to traverse the host surface through reversible interactions that are sufficiently weak to permit detachment of one or two fibers, but strong enough to prevent complete desorption.

The two-step model describing phage adsorption has been widely used as a standard framework for characterizing this RBP-receptor interaction (reversible interaction which led to irreversible attachment) (Storms and Sauvageau, 2015). In 1952 Stent and Wollman proposed three hypotheses explaining the two-step model: the activity–inactivity model, the competitive model, and the sequential model (Stent and Wollman, 1952). Subsequent studies of T4 adsorption kinetics concluded that the sequential model was the most appropriate. The sequential model has been applied to numerous other phages than T4, such as T1, T2, T4, T5, λ, etc. Nevertheless, it is now recognized that the applicability of a particular model describing this interaction depends not only on the bacteriophage itself but also on the surrounding environmental conditions. Another important aspect is the heterogeneity of phage population, which can likewise be categorized into fast and slow adsorbers (biphasic model). This is mainly attributed to mutations or structural defects in tail proteins, which influence the efficacy of host recognition. However, the suitability of a particular model is determined by the phage under investigation, the environmental conditions, and the intended application (Zonenstein et al., 2010; Storms and Sauvageau, 2015).

Phage adsorption is governed by RBPs, which mediate the initial contact with bacterial hosts. Therefore, understanding the phage adsorption mechanism, requires detailed investigation of RBPs' structure and function. At the same time, applied research contributes to a deeper understanding of the phage-host interactions. Studies on RBPs provide molecular insight into adsorption efficiency, host specificity, and phage infectivity. Moreover, they enable the engineering of phages with altered host ranges for therapeutic and biotechnological applications.

**Figure 1:**
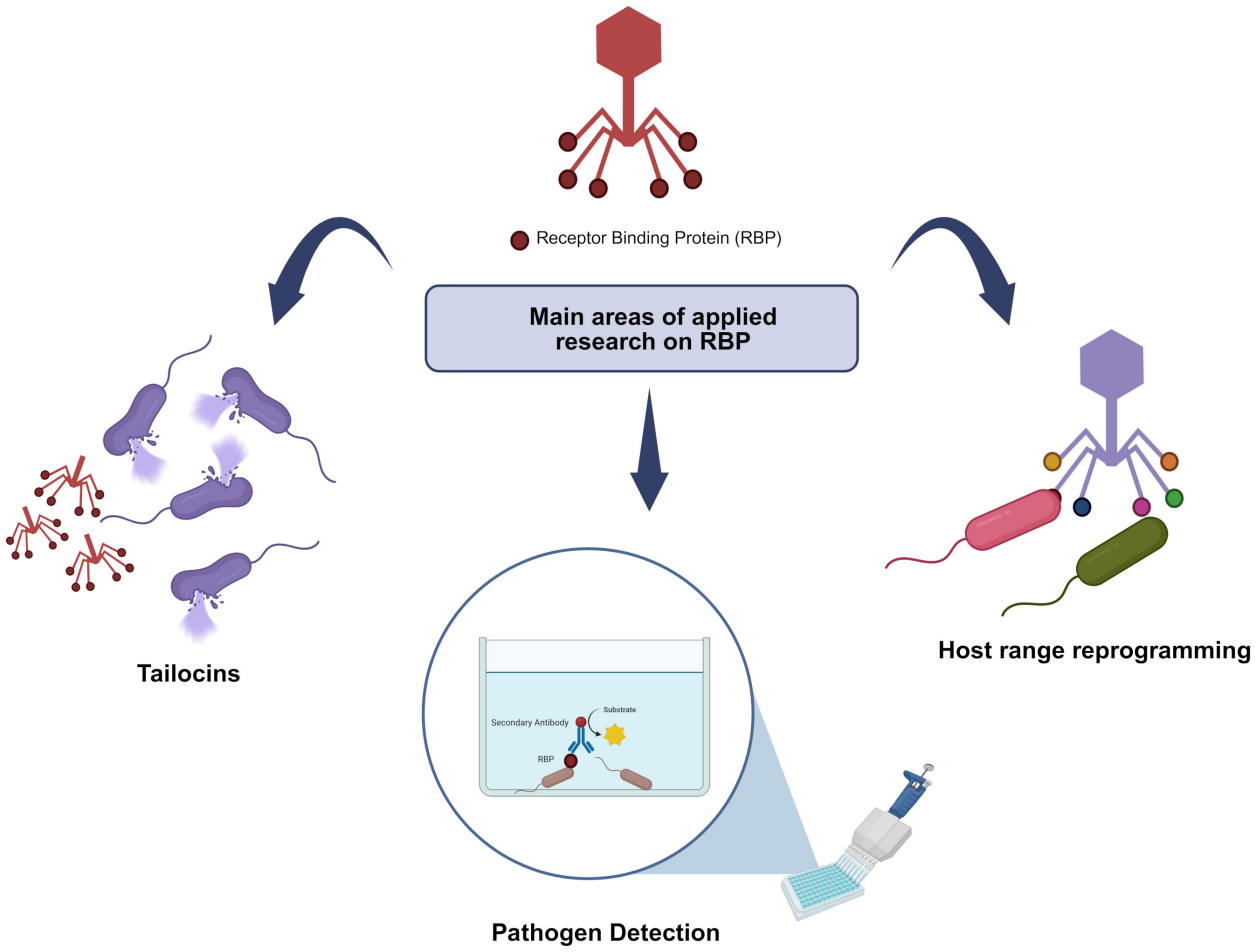
The overview of the applied research on bacteriophage Receptor Binding Proteins (RBPs). Currently, the use of RBPs for the diagnosis and detection of pathogens is attracting the most attention. Host range reprogramming is an interesting approach that could enhance the efficacy of phage therapy. The ability of a phage to infect bacterial host is largely governed by its tail structures and RBPs, which mediate recognition of specific surface receptors, and then irreversible attachment. Therefore, significant research has focused on extending phage host ranges by applying directed evolution strategies and rational, structure guided engineering of tails as well as RBPs. Tailocins represent an additional antibacterial promising strategy and are of particular interest in the context of plant pathogens. Tailocins are high molecular weight bacteriocins that enable antagonistic interactions among closely related bacterial strains. They are derived from bacteriophage tail structures, but they lack of capsid. Created with BioRender.com

In recent years, RBPs have gained significant attention as important research targets. This trend is largely driven by the growing problem of antibiotic resistance, which has intensified efforts to identify alternative strategies for pathogen eradication. Consequently, considerable interest has been devoted to endolysins and depolymerases. The activity of this enzymatic proteins leads to bacterial lysis or degradation of external structures such as capsules. Thereby weakening pathogens and facilitating their elimination by the immune system or conventional therapeutics. Currently, RBPs are undergoing a renaissance. Once underestimated, they are now the focus of numerous studies. Both in terms of their use in pathogen diagnostics, as tools for improving phage therapy through host range programming, and tailocins, protein-based antibacterial weapons to kill or inhibit the growth of bacteria (Figure 1). More studies in this area can facilitate the development of phage-based therapeutics.

**Figure 2:**
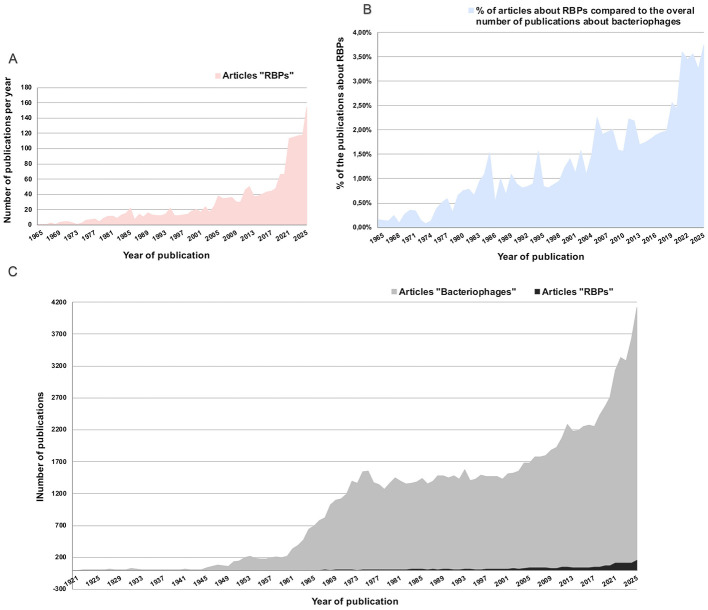
Timeline represents **(A)** increasing number of articles about RBPs, **(B)** increasing % of the RBPs articles compared to all publications on bacteriophages, and **(C)** comparison of the number of articles on bacteriophages and on RBPs alone. The figure was prepared with the help of the PubMed database using the following data search scheme: (bacteriophage[MeSH Terms] OR bacteriophage*[Title/Abstract] OR phage*[Title/Abstract]) AND (RBP[Title/Abstract] OR receptor-binding[Title/Abstract] OR receptor binding[Title/Abstract] OR host-binding[Title/Abstract] OR host bindingTitle/Abstract] OR adsorption protein[Title/Abstract] OR attachment protein[Title/Abstract] OR adsorption apparatus protein[Title/Abstract] OR adsorption apparatus[Title/Abstract] OR “tail fiber”[Title/Abstract] OR “tail fibers”[Title/Abstract] OR “tail spike”[Title/Abstract] OR “baseplate protein”[Title/Abstract] OR “distal tail protein”[Title/Abstract] OR “tail tip protein”[Title/Abstract]).

The primary aim of this review was to systematically assess the evolution and current scope of research interest in bacteriophage RBPs. This publication analyzes trends over time and evaluate their significance within the broader field of bacteriophage biology. To achieve this, we performed a focused analysis of the available literature, tracking the emergence and growth of RBPs-related studies. The first reports about bacteriophages appeared around 1920.Whereas studies specifically addressing RBPs and other structural proteins began to emerge between 1960 and 1970. These early works primarily focus on identifying genes responsible for tail fiber structure and elucidating the mechanism of bacterial adsorption. Using the PubMed Advanced Search Builder, we performed a brief analysis of the available literature records up to the year 2025. The search was designed to identify publications related to bacteriophages and their host recognition machinery. The first query block captures all bacteriophage-related literature by combining a MeSH controlled vocabulary term with free-text truncated keywords (bacteriophage^*^, phage^*^) searched in titles and abstracts. The second block targets specific molecular structures responsible for bacterial host recognition and adsorption, including RBPs, attachment proteins, and adsorption apparatus components. It also encompasses structural elements of the phage tail, such as tail fibers, tail spikes, baseplate proteins, distal tail proteins, and tail tip proteins. The two blocks are joined by the Boolean operator AND, ensuring that only publications addressing both bacteriophages and their host-binding structures are retrieved. No filter was applied to exclude review articles, and no language restriction was applied during the search. A PubMed search identified 1,633 records related to RBPs. In recent years, a marked increase in bacteriophage research has been observed, particularly in studies focused on RBPs. In 2025, the number of RBPs-related publications reached a peak of 154 articles. Although the number of papers containing RBPs-related terms in their abstracts has increased steadily year by year, they still account for less than 4% of all bacteriophage-related publications. All analyzed articles were directly related to bacteriophage research, and numerical data were presented at Figure 2.

Overall, these findings highlight both the dynamic expansion of RBPs focused research and the need for continued, more integrated studies to fully elucidate the roles and applications of RBPs in bacteriophage biology. Simultaneously, they underscore the significant application potential of RBPs. Particularly in areas such targeted antibacterial therapies, diagnostics, and bacterial detection systems, where their high specificity for host receptors can be effectively exploited.

## A bacteriophage Receptor Binding Proteins based approach to pathogen detection

The primary and most extensively investigated aspect of the practical use of phage RBPs concerns pathogen recognition. This field of research focuses on the mechanisms underlying phage attachment to bacterial hosts. The reviewed studies suggest that phage proteins are gaining significance as diagnostic tools. Due to the discovery of novel phages as well as the growing understanding of the specificity and functional roles of phage tail proteins. The interactions between phages and their hosts are influenced by phage morphology, bacterial surface structures, and environmental factors. Monovalent bacteriophages infect only a specific bacterial strain, whereas polyvalent bacteriophages can target multiple bacterial strains (Filik et al., 2022). The structural heterogeneity of bacterial cell envelopes results in a diverse repertoire of potential receptor molecules for phage recognition. In Gram-positive bacteria, cell wall components such as teichoic acids and lipoteichoic acids frequently serve as primary binding sites. Whereas in Gram-negative bacteria, receptor structures typically include lipopolysaccharides (LPS), outer membrane proteins (e.g., porins), as well as surface appendages such as pili and flagella. A substantial number of reviews and studies have highlighted host-associated receptors that interact with bacteriophage RBPs. Receptors recognized by bacteriophages usually include a primary receptor for reversible attachment and a secondary receptor for irreversible attachment. The final receptors are mainly proteins or cell wall polysaccharides (Koç et al., 2016).

The use of RBPs in pathogen detection is primarily focused on the development of highly specific and relatively rapid methods that, in some cases, can replace classical diagnostic approaches. These conventional methods are still largely based on culture or biochemical techniques, which often fail to provide clear results. Therefore, the use of proteins characterized by high binding specificity appears to be well justified in this context. In our previous paper by Filik et al. (2022) we presented various approaches in which phage proteins were adapted and employed to improve pathogen diagnostic processes, mainly by achieving high specificity. Additionally, methods characterized by a high level of pathogen detection sensitivity were described, which in some cases significantly exceeds that of classical diagnostic techniques (Filik et al., 2022). The methodologies for the detection of pathogens such as *Acinetobacter baumannii, Campylobacte*r spp., *Yersinia pestis, Pseudomonas aeruginosa, Listeria monocytogenes, Staphylococcus aureus, Enterococcus* spp., *Salmonella* spp., and *Shigella* were shown. These pathogens can be identified using capture-based detection approaches, including phage protein–coated nanoparticles, ELISA (Enzyme-Linked Immunosorbent Assay) techniques, or biosensor-based methods. Since 2022, based on PubMed searching, 9 papers considering pathogen diagnostics using RBPs have been published (Table 1).

**Table 1:** Diagnostic tools based on bacteriophages RBP since 2022.

Lp.	Pathogen	RBP (bacteriophage)	Used methodology	Limit of detection (if applicable)	References
1	Shiga toxin-producing *Escherichia coli* (STEC)	pO91-ORF43, pO103-ORF42, and pO111-ORF8 (pO91, pO103, and pO111)	Indirect enzyme-linked immunosorbent	33 CFU/mL	Chen et al., 2023
2	*Escherichia coli*	gp17 (T7)	GFP-tagged TFP (GFP-TFP) and fluorescence microscopy. TFP conjugated with magnetic beads. Colorymetric [substrate chlorophenol red-β-D-galactopyranoside (CPRG)]	10^2^ CFU/mL	Hong et al., 2023
3	*Salmonella*	long-tail-fiber proteins (LTF4-a)	magnetic nanoparticles (MNPs)	7 CFU/mL	Wang et al., 2022
4	*Listeria monocytogenes*	TFP (gp99) (phage A511)	dual recognition elements: phage TFP and triple-functional nanozyme lateral flow biosensor	10 CFU/mL	Pan et al., 2023
5	*Escherichia coli* O157:H7	TFP:gp13 (phage EP335)	proteins-nano magnetic beads (PBPs-MBs)	2.44 × 10^2^ CFU/mL	Hong et al., 2024
6	*Acinetobacter baumannii*	gp50 (phage Abp9)	Fluorescence emission	2.0 × 10^2^ CFU/mL	Chen et al., 2022
7	*Vibrio cholerae*	ORF93 (phage ICP1 )	-	-	Sayeed et al., 2025
8	*Citrobacter koseri*	LTF protein (gp267) (CkP1 Phage)	Fluorescence microscopy, Biacore	-	Oliveira et al., 2023
9	*Salmonella* pullorum	TFP 35Q (phage YSP2)	ELISA	-	Deng et al., 2024

## Host-range reprogramming as a tool for broadening phage specificity

Host-range reprogramming represents another compelling area of phage therapy research. Phage reprogramming is mostly associated with tail machinery and RBPs, which recognize molecules (receptors) exposed on bacteria surface and leading to irreversible attachment and genome release. Naturally occurring lytic phages often have narrow host ranges, which sometimes limit their therapeutic potential. As a result, there is substantial interest in expanding these ranges through directed evolution or structure-guided engineering of their tails and RBPs. Additionally, bacteria can quickly become resistant to phage infection by altering their surface molecules through spontaneous mutation or phenotypic variation. This can lead to a bottleneck when using phages as a therapeutic (Dunne et al., 2021). One solution is to use phage cocktails of multiple unrelated phages that have different host ranges and target different surface receptors, thereby reducing selective mutation pressure on any single receptor. Despite these advantages, this solution also has some limitations. The formulation and production of these coctails can be time-consuming, however, in life-threatening infections, rapid intervention is crucial.

Over the past decades, high-resolution models of diverse phage tail architectures have become available, providing a structural framework for engineering RBPs to modulate host range. Historically, RBP has relied primarly on two structure based strategies: (i) domain swapping and (ii) structure-guided mutagenesis (Ando et al., 2015; Dunne et al., 2019). The domain-swapping approach exploits the modular nature of RBPs to generate chimeric proteins in which receptor binding domains are exchanged to enable recognition of alternative hosts. In parallel, structure-guided mutagenesis conceptually similar to antibody engineering introduces targeted variability into RBP binding sites, generating libraries of mutants with altered ligand specificities (Liu et al., 2002; Saeed et al., 2017; Dunne et al., 2019, 2021). Both strategies are mostly reliant on the structure. For example, swapping domains can be accomplished with minimal understanding of receptor binding or the potential enzymatic capabilities of an RBP. Similarly, random mutagenesis can give the same results, but the major disadvantage will be the lack of control over where the mutation will occur (Dunne et al., 2021).

The field has subsequently evolved and Dunne et al. (2021) proposed four strategies for precise RBPs engineering (Table 2). The long-used and easiest approach is non-targeted recombination of phage genomes. Several rounds of incubation of phage cocktails with susceptible and resistant bacterial strains produce phages with different ranges based on the adaptation mechanism to the host. In addition, there are three strategies that are more directed and based on the molecular biology protocols. Modification of host-range can be achieved through homologous recombination of RBP genes or the electroporated phage genome with the cytosolic RBP template encoding the desired host range, enabling tail fiber exchange (trading tail fibers by directed homologous recombination). For example, this approach was used to expand the host range of T4 and T2-like phages (Tétart et al., 1998; Yoichi et al., 2005). The homologous recombination approach is limited by the need for high sequence similarity between the two RBPs genes to facilitate recombination. The next method is the complete replacement of the tail which allows for the exchange of RBP and tail components between different phages. The approach relies on the synthetic biology platforms and offers the production of the phages from the individual fragments. The last but most accurate method is directed mutagenesis of TF binding loops. In many cases, C fragments of TF are responsible for binding. Any mutations can lead to the generation of new phage populations with different or expanded host ranges. All four strategies are presented in Table 2.

**Table 2:** Strategy for RBP engineering (Dunne et al., 2021).

Strategy	Principle	References
Non-targeted recombination of phage genome	Cycling a cocktail of phages with a group of susceptible and resistant bacteria until a recombinant phage appears.	Burrowes et al., 2019
Trading tail fiber directed homologous recombination (HR)	A more direct approach to modify the host range is to only recombine the RBP gene(s) of an infecting phage or electroporated phage genome with a cytosolic RBP template featuring the desired host range. The limitation of the approaches is that the two RBP genes must feature high sequence similarity to allow recombination.	Tétart et al., 1998
Complete replacement of tail components	Ando et al. developed a yeast-based platform to produce synthetic phage genomes assembled from individual fragments (exchange of RBP and tail components between different phage backbones). An alternative platform for producing synthetic Gram-Positive targeting phages was developed by Kilcher et al. for rebooting *in vitro*-assembled synthetic genomes in *Listeria* L-forms	Ando et al., 2015; Kilcher et al., 2018
Directed mutagenesis of tail fiber binding loops	Directed randomization of the distal loops of tail fibers can generate phages with new host ranges.	Trojet et al., 2011

An excellent example of host-range reprogramming is the VersaTile platform that serves as a precise tool for tailocin engineering, enabling rapid and straightforward integration of RBPs from diverse origins. The platform evaluated tailocins equipped with various RBPs targeting O-antigens or K-antigens of *Escherichia coli* and *Klebsiella pneumoniae*. Researchers demonstrated that eight tailocins were redirected toward the O-antigen of *E. coli*, one toward the *E. coli* K-antigen, and three toward the K-antigens of *K. pneumoniae*. Additionally, a bivalent R2 tailocin targeting K-antigens of both *K. pneumoniae* and *E. coli* was proposed and experimentally validated through determination of the specificity spectrum (Dams et al., 2025).

A significant advance was reported by Dunne et al. in 2019. They presented the first engineering of a Gram-positive targeting phages using the PSA *Listera* phage as a model. The first strategy used by the authors was RBP-directed mutagenesis, which enables directed evolution and phage-host coevolution by increasing the mutation rate in a gene-specific manner. Their findings provided evidence that a single amino acid substitution is sufficient to alter RBP specificity toward closely related receptors. A second approach involved the construction of a polyvalent phage encoding both wild-type and mutated RBPs within a synthetic PSA phage genome. The final approach consisted of solving the crystal structure of Gp15, which providing a blueprint for structure-guided design of phages with chimeric RBPs and altered host specificity (Dunne et al., 2019).

According to the accumulated data the key steps required for the successful development of engineered phages are as follows: (1) identification of host-range determining regions; (2) introduction of genetic variability; (3) screening of activity against bacterial strains; (4) evaluation of the response to engineered phages, including comparison with naturally evolved phages; and (5) assessment of the potential development of bacterial resistance, as well as analysis of phage–bacteria interactions (Jia et al., 2023).

## Tailocins–killer tails

Another direction of the research on RBPs is tailocins. Tailocins are promising and attractive alternative antimicrobials and have already been proven effective against several pathogenic bacteria (Table 3) with no or few side effects on mammalian cell lines (Woudstra et al., 2024). They are high-molecular-weight bacteriocins that can mediate antagonism between related bacteria (to kill competitors by binding their target) as well as interaction with eukaryotic cells. Tailocins reassemble the tail structure of bacteriophages and do not include proteins that build the capsid (Ghequire and De Mot, 2015). They can be divided into two groups: contractile (R-type) and non-contractile (F-type) tails. R-type tailocins consist of a tube surrounded by a contractile sheath linked to a baseplate structure with a RBP attached, whereas F-type tailocins consist of a tube without a sheath that contains a baseplate where the RBP attaches. R-type tailocins can lead to the death of targeted bacteria by puncturing the bacterial membrane. They bind to the bacteria through RBPs on the distal part of the tail fibers. The RBPs' attachment triggers the contraction of the sheath, and the tail tube perforates the bacterial membranes, resulting in the dissipation of the membrane proton-motive force, which promotes essential movement of the protons across the membrane. The main advantage of the R-type tailocins is that they kill bacteria without damaging the cell envelope integrity. The R-type tailocin gene cluster is a common feature of several strains of Gram-negative (often plant-associated) (Patz et al., 2019) bacteria. R-type tailocis have been used *in vivo* in a mouse model to treat *Pseudomonas aeruginosa* infection with success (Six et al., 2021). It is assumed that F-type tailocins work similarly, but the detailed mechanism of action is still unknown. To date, three principal strategies have been employed for tailocin production: (I) disrupting the capsid-tail junction of phage particles, (II) blocking capsid assembly during phage propagation, and (III) creating headless phages using synthetic biology tools (Woudstra et al., 2024) (Table 4).

**Table 3:** Tailocins with proven effectiveness against pathogenic bacteria.

Tailocin	Targeted bacteria; disease	Method of obtaining	References
Engineered R2 tailocins with a chimeric RBP (cross-species bivalent tailocin R2-K11-K1)	*Escherichia coli* K1 *Klebsiella pneumoniae* K11	Using VersaTail technique for the assembly of non-homologous sequences of modular proteins such as RBPs	Dams et al., 2025
Burkholderia multivorans WS-FJ9 tailocin (Bm_67459 belonges to the phage TAC_7)	*Phytophthora cinnamomi*	Construction of a prokaryotic expression vector with the phage tail-like protein	Wen et al., 2025
Pragia fontium 24613 tailocin	Clinically significant enterobacteria, including *Yersinia enetrocolitica*	Like in phage protocols—with centrifugation, precipitation in PEG and CsCl gradient purification	Snopková et al., 2025
Campycins produced by *Psudomonas aeruginosa* (campycin 1 and campycin 2)	*Campylobacter jejuni*	Expression and purification were conducted using *P. aeruginosa* PAO1 with an ultracentrifugation step	Zampara et al., 2024
BglaTNB6 tailocin from *Burkholderia gliadoli* NB6	Rice seed-borne disease caused by *Burkholderia glumae* and *Burkholderia plantarii*	Tailocin/phage purification method, including ammonium sulfate precipitation and ultracentrifugation	Kouzai et al., 2024
Rhizoviticin F-type tailocins	*Allorhizobium vitis*	The bacterial culture was exposed to UV, treated with benzonase nuclease, centrifuged, precipitated with PEG, and fractioned with gel filtration.	Ishii et al., 2024
Diffocins–Avidocin-CDs (Av-CD291.1, Av-CD-291.2)—R-type bacteriocins from *Clostridium difficile*	Competing *Clostridioides difficile* strains including highly pathogenic 027 ribotype, including hypervirulent BI/NAP1/027 strain (tested *in vivo*)	Cloning diffocin gen clusters in *B. subtilis*, using hydrogen peroxide to induce diffocin expression, PEG and ultracentrifugation to concentrate and purify	Bratkovič et al., 2024; Gebhart et al., 2015
*Salmonella* phages tailocins (S117, Fels-1, Fels-2, Gifsy-1, Gifsy-2)	*Sallmonella typhimurium*	Genetic modification of virulent *Ackermannviridae* phage S117, phages Fels-1,-2 and Gifsy-1,−2 using CRISPR-Cas9 and deleting major capsid gene	Woudstra et al., 2023
Pantailocins (Tailocins from *Pantoea ananatis* and *Pantoea stewartii subsp. Indologenes*)	*Pantoea*, phytopathogens	Cell culture with mitomycin C, PEG precipitation	Stice et al., 2023
Dickeyocin P2D1 (phage P2-like dickeyocin 1)	*Dickeya* spp., soft rot disease	Cells of *D. dadantii* 3937 treated with mitomycin C	Borowicz et al., 2023
*Pseudomonas syringae* USA011R tailocins	*Salmonella enterica Erwinia amylovora, Xanthomonas perforans*	Cells treated with mitomycin C, chloroform treatment, PEG precipitation	Weaver et al., 2022; Baltrus et al., 2022
*Pseudomonas fluorescence* SF4c produced tailocin	*Xanthomonas* (*X. axonopodis pv vesicatoria* Xcv Bv5-4a); bacterial-spot disease in the tomato	*P. fluorescence* SF4c treatment with mitomycin C, ammonium sulfate precipitation and centrifugation	Fernandez et al., 2017; Príncipe et al., 2018

**Table 4:** Three strategies for creating tailocins (Woudstra et al., 2024).

Lp	Strategies	Method of creating tailocins
I	Disrupting the capsid-tail junction of phage particles	Osmotic shock treatment of phage particles—employing cesium chloride as an osmotic shock buffer (Coombs and Eiserling, 1977)
Chemical inactivation of phage particles – using commercial sanitizers based on quaternary compounds (Hayes et al., 2017)	
II	Blocking capsid assembly during phage propagation	Multiple infection of bacterial cells by phages—using a high ratio of phage particles per bacterial cell (Pickard et al., 2010)
Genetically engineering virulent phages into tailocins—using CRISPR-Cas approaches for deleting genes essential for capsid synthesis (Williams et al., 2008)	
Genetically engineering virulent prophages into tailocins—deleting genes essential for capsid synthesis or assembly (Woudstra et al., 2023)	
III	Creating headless phages using synthetic biology tools	Using synthetic biology to rebot phages into a tailocin-plasmid—by genetically removing the capsid gene (Pfeifer et al., 2021)
The cell-free expression system as a tailocin factor—cell-free extracts provided with DNA from phage genomes lacking capsid genes, a tailocin-plasmid, or a DNA fragment containing the genes for the phage tail synthesis (Emslander et al., 2022)	

There is also another division of the Tail-like related structures known in the literature, which in fact can be called Contractile Injection Systems (CISs) and can be divided into two groups: (I) intracellular, cell wall-anchored type VI secretion systems (T6SSs) and (2) extracellular CISs (eCISs). They are both associated with the pathogen virulence. The eCISs comprise the R-tailocins produced by various bacterial species (Heiman et al., 2023). The name is based on the fact that these contractile structures are released into the extracellular environment upon suicidal lysis of the producer. The best-known CISs are the T6SSs, which act primarily against bacteria but are also involved in interactions with eukaryotic cells. The bacterial type VI secretion system is an organelle that is structurally and mechanistically analogous to an intracellular membrane-attached contractile phage tail. T6SSs are intricate syringe-like complexes docked into the cytoplasmic membrane of the attacking bacteria, which inject toxic and lytic effectors into target cells. These effectors disrupt basic cellular components, including the cell wall, cytoskeletal structures, and nucleic acids. In addition to directly targeting eukaryotic cells, the T6SS can also target other bacteria coinfecting a mammalian host, highlighting the importance of the T6SS not only for bacterial survival in environmental ecosystems but also in the context of infection and disease (Ho et al., 2014).

The widespread occurrence of tailocins across phylogenetically diverse bacterial genera suggests that these particles contribute significantly to bacterial fitness in various ecological niches. The tailocins are very often produced by Soft Rot Pectobacteriaceae (SRP) bacteria. Plant-pathogenic SRP, comprising *Pectobacterium* spp., *Dickeya* spp., and *Musicola* spp. (formerly classified as pectinolytic *Erwinia* spp.), represent a useful model for investigating the environmental role of tailocins. Due to the wide range of environments inhabited by SRP bacteria, these pathogens are likely to encounter diverse microbial competitors with which they must effectively compete. One of the examples is Dickeyocin P2D1—induced and isolated from the pectinolytic *Dickeya dadantii* strain 3937 (Borowicz et al., 2023). Cells of *D. dadantii* 3937 treated with mitomycin C produced syringe-like macromolecular structures resembling bacteriophage tails and are phylogenetically related to the tail of *Peduovirus* P2. P2D1 could kill a variety of different *Dickeya* spp. but not *Pectobacterium* spp., and were not toxic *to Caenorhabditis elegans*. This study demonstrated that P2D1 killing efficiency is bacterial species dependent and showed that tailocin directly punctures the cell envelope, leading to fast killing—in some cases even after 20 min of incubation. Of course, additional studies are needed to fully explore the potential of dickeyocin P2D1 to achieve plant disease control, but the evidence clearly state that P2D1 could be used in controlling SRP infections in crops (Borowicz et al., 2023).

Natural and engineered tailocins represent two distinct yet complementary approaches to targeted antibacterial interventions. Naturally occurring tailocins are produced by bacteria as competitive weapons and have evolved to eliminate closely related strains occupying the same ecological niche. Their antibacterial activity is typically characterized by a high degree of specificity, reflecting long-term adaptation to particular bacterial surface receptors. While such specificity enables precise targeting with minimal disruption of non-target microbiota, it may also limit their effectiveness against a broader range of clinically relevant pathogens. In contrast, engineered tailocins capitalize on advances in synthetic biology and RBPs engineering to modify or expand their target spectrum. Through the replacement or rational design of RBPs and associated tail components, engineered tailocins can be redirected toward alternative bacterial receptors, thereby overcoming some of the host range constraints of naturally occurring systems. Consequently, engineered tailocins constitute a versatile platform for the development of next-generation precision antimicrobials with customizable target specificity.

Since tailed phages and phage tail-like particles share the ability to penetrate bacterial cell walls, both could be applied as pharmaceutical or biocontrol agents against pathogenic bacteria. The application of tailored or even engineered biocontrol bacteria possessing specific tailocins for antagonizing plant pathogens is a challenging goal for the future. The interaction between bacterial cell surface receptors and RBPs on tail fibers is extremely complex. Suppose one manages to find tight correlations between tail fiber genes and cell surface receptors of target bacteria. In that case, this would pave the way for selecting appropriate candidate bacteria from strain collections and molecular engineering of tailocins as a promising tool to combat pathogens (Patz et al., 2019). Practical application of tailocin is favorable due to their many advantages. One of the main advantages of tailocins is that they cannot replicate at the site of application. Similarly, they cannot spread through transduction or transformation, since only the protein complexes, not the encoding genes are used. Their high target specificity also prevents disruption of non-target, often beneficial, bacteria in the same niche (Borowicz et al., 2023). On the other hand, one might also argue that inability to replicate at the site of infection is a disadvantage, as tailocins may be more easily inactivated than whole phages due to their non-renewable nature.

## Toward tail fiber proteins structure atlas—RBPseg

**Figure 3:**
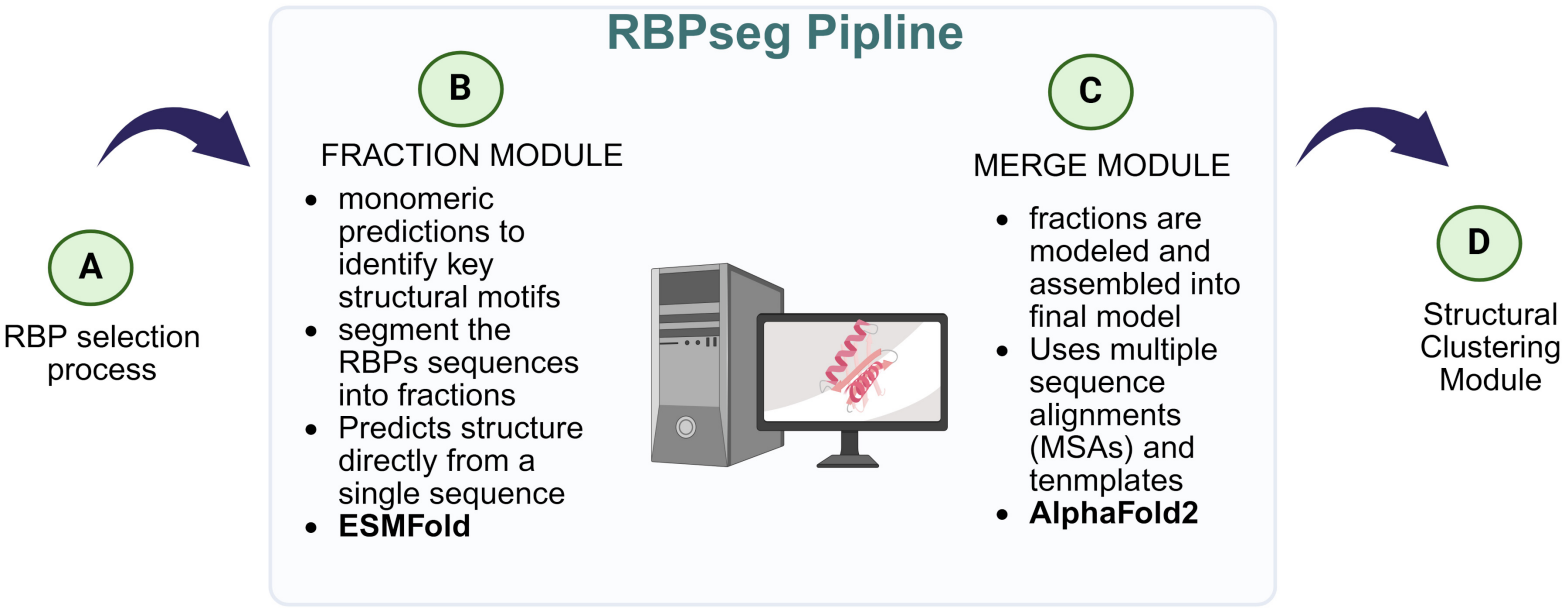
RBPseg pipeline for the RBP structure prediction, which can help build the atlas of RBPs. The pipeline consists of 4 steps: **(A)** RBP selection process; RBPseg is an implementation of two protein structure prediction approaches, ESMFold [**(B)** Fraction Module] and AF2M [**(C)** Merge Module], to handle large fiber-like multimeric structures, and finally **(D)** Structural Clustering Module. The tool was designed to improve the predictability of the full quaternary structure of phage tail fibers and spikes. This breakthrough could greatly enhance phage biotechnology, paving the way for applications such as engineered phage therapeutics. Nevertheless, current approaches to protein complex modeling are still challenged by the elongated and flexible nature RBPs. Created with BioRender.com.

Thanks to the development of advanced research methods, and the growing interest in how RBPs work, more research branches are emerging that explore these proteins, which are key to phage infection. Klein-Sousa et al. (2025) demonstrates the potential of constructing an atlas of tail fiber proteins (RBPs) using RBPseg pipeline (Figure 3), which can improves RBP structures prediction by increasing the confidence of AlphaFold 2 models (Klein-Sousa et al., 2025).

The structural atlas of RBPs serves as a valuable resource for studying how phages have adapted their recognition machinery which was built with the help of RBPseq. The first tail fiber structural atlas was created by using the structural module in combination with hidden Markov models with its covering 24% of a set of pre-annotated tail fibers on UniProtKB. This major step and could facilitate phage biotechnology applications, such as custom-designed phage therapies. Current experimental approaches for modeling protein complexes face significant limitations, particularly when dealing with elongated and flexible RBPs. Single-phage cryo–electron microscopy often struggles to produce high-resolution maps due to substantial conformational variability. Similarly, obtaining high-quality crystals of full-length fibers poses a major challenge for structural determination using X-ray crystallography. Despite these experimental obstacles, recent advances in computational protein structure prediction such as AlphaFold, ESMFold, and AlphaFold2-multimer (AF2M) have made it increasingly feasible to generate reliable models of proteins and protein complexes. Modeling large protein complexes using standard AF2M pipelines still faces computational limitations. Primarly due to hardware and memory issues, resulting in models with large low-confidence regions, clashes, and long computing times. Strategies to address the challenge of building large complex models proposed, but were not specifically designed for RBPs (Klein-Sousa et al., 2025). Previous studies have used AF2M to predict RBPs structures, relying on expert-driven manual segmentation of sequences into smaller fragments, modeling each fragment separately, and then assembling them into a final structure (Cambillau and Goulet, 2023; Pas et al., 2023). However, a fully automated, systematic approach for identifying both the positions and the required number of segmentation points remains lacking. Introduced by the authors, pipeline RBPseg identifies major domains and segmentation hotspots in tail fibers by analyzing their primary structure with ESMFold and a sigmoid distance-pair (sDp) function (Klein-Sousa et al., 2025). It then predicts tail fiber segments in parallel using AF2M and assembles complete fiber models. This integrated workflow overcomes computational limitations, enhances performance, and produces more reliable models for large tail fibers. Overall, this method offers a robust framework for predicting, classifying, and characterizing phage tail fibers. This could provide new insights into their structural features and potential functions. The authors used RBPseg for selected model phages and classified the predicted RBPs based on structural similarity and domain specificity. These models account for 24% of the pre-annotated tail fibers in UniProtKB. They offer valuable insights for analyzing the phage toolkit for host attachment by revealing structurally related domains (Klein-Sousa et al., 2025). To sum up, developing reliable models of all potential RBPs will not only enhance our understanding of phage-bacterial coevolution but also nurture biotechnological and biomedical applications of phages, such as custom-designed phage therapy (Klein-Sousa et al., 2025).

## Innolysins—combine the activity of endolysins with the binding capacity of RBPs

Another innovative strategy involves integrating the mechanisms of action of endolysins and RBPs. Endolysins are enzymes that degrade bacterial peptidoglycan, and native endolysins have been successfully applied as antibacterial agents against Gram-positive bacteria, including *Staphylococcus aureus* and streptococci. However, the outer membrane of Gram-negative bacteria generally prevents endolysins from accessing the peptidoglycan layer, limiting their effectiveness against these pathogens. To enable the killing of Gram-negative pathogens using endolysins, several engineering strategies have been proposed, one of which involves the development of Innolysins. Innolysins were developed by Zampara et al. (2020) through the fusion of the phage T5 endolysin and the phage Pb5 RBP in different configurations. In their study, the authors exploited the binding specificity of the phage T5 RBP to its cognate FhuA receptor to enable an endolysin to exert antibacterial activity. To identify variants with high antibacterial activity, a library of 228 Innolysins was generated. Obtained results demonstrate that Innolysin Ec6, similar to bacteriocin-derived fusions with endolysins, can target isolates of bacterial species other than *E. coli*, including *Shigella sonnei* and *Pseudomonas aeruginosa*, in the presence of receptor FhuA. Notably, Innolysin Ec21 exhibited bactericidal activity against third-generation cephalosporin resistant *E. coli*. Overall, the Innolysin strategy represents a significant expansion of previous endolysin engineering approaches (Zampara et al., 2020).

## Combining properties of antibodies and RBPs

One approach currently used in the development of antibacterial therapies is the use of bacteriophage proteins. Some researchers see huge potential in RBPs due to their unique properties. This enables them to bind receptors on the surface of bacteria, in addition to other described characteristics. In a CellPress article, Swanson and Cingolani, based on the article by Dunne et al. (2018) show that important findings: a high structural and functional similarity between the variable regions of RBPs and the hypervariable regions of antibodies. These are proteins that evolved for completely different purposes, but due to their functions, i.e., binding specific targets with high specificity, they exhibit great functional similarity. The two types of recognition molecules share strikingly similar design principles. In both the polyglycine-rich PGII sandwich (in S16 phage adhesion) and the Ig domain, a relatively flat structure built from short Gly-rich helices or β-strands supports several surface-exposed loops (five in PGII, three in each Ig domain) at one end of the molecule. These loops act like “fingers” extending from a “palm,” continually probing surfaces for their specific targets. Because the PGII and Ig folds are flat, their antigen-binding loops can be packed closely together, which enhances binding through avidity. Both adhesins and immunoglobulins are also highly adaptable, capable of evolving new recognition features. This is reflected in the pronounced variability of the loops in the PGII sandwich and the hypervariable complementarity-determining regions (CDRs) in Igs (Dunne et al., 2018; Swanson and Cingolani, 2018). In our laboratory, we used these finding to study the possibility of combining the properties of antibodies and RBPs. In the paper Filik-Matyjaszczyk et al. (2025), we studied the possibility of the opsonophagocytosis of RBP fused with the Fc fragment of IgG1 (Fc_RBP). We proved that the bifunctional protein can improve phagocytosis of bacterial cells opsonized by the proposed Fc_TFPgp17 fusion protein in an *in vitro* model (Filik-Matyjaszczyk et al., 2025). To date, this represents the first report of an RBP fused to an antibody fragment to facilitate pathogen clearance by immune system cells. A comparable strategy has been described only for endolysins, where cell wall binding domains derived from endolysins were fused to antibody Fc fragments (Raz et al., 2017).

## Summary

In 2025, the highest number of publications on RBPs was published (154 records), reflecting the growing interest in RBP-based technologies. RBPs possess properties that are highly desirable for pathogen recognition, indicating their strong potential for improving the detection and control of pathogenic bacteria. Compared with conventional microbiological methods, RBP-based diagnostic strategies may enhance traditional workflows by simplifying and accelerating diagnostic procedures while eliminating the need for bacterial culture.

Another relevant research area is host range reprogramming. Although phage therapy has been known in science and clinical practice for over 100 years, it has not yet achieved widespread use despite its many advantages. It remains largely an experimental therapy, applied mainly in life-threatening cases or when all other therapeutic options have failed. Advances in molecular and synthetic biology may help meet the regulatory and technical requirements for phage-based therapeutics. Introducing targeted modifications such as host range reprogramming could not only enhance our understanding of phage therapy but also facilitate its broader and more effective implementation. In addition, host range reprogramming may allow better control over the pharmacokinetics of phages administered to patients. Obviously, engineering phage RBPs offers promising route to tailor phage host range, but structural characterization of these structurally complex protein continues to limit the progress. The RBPseq platform described earlier is expected to significantly enhance our knowledge of RBPs architecture. Nevertheless, while *in silico* approaches provide valuable predictions, many of presented structures will still require experimental validation. Another issue that should be taken under consideration is the potential of acquiring resistance to engineered phages in the similar manner as it is observed with wild type phages. Additionally, as well as with wild type phages, engineered phages are capable of inducing anti-phage antibody response, which also may limit their effectiveness during therapy.

A distinct branch of research focuses on tailocins. Similar to endolysins, tailocins act as antibacterial agents by disrupting bacterial cell membranes and inducing pore formation upon contact with target cells. Their application may be considerably simpler than that of whole bacteriophages. As protein complexes, tailocins offer a notable advantage: they do not transfer genetic material, thereby eliminating the risk of horizontal gene transfer. Moreover, as molecules with a well-defined qualitative and quantitative composition, they may be easier to standardize, validate, and regulate for therapeutic use or for industrial applications, such as plant protection products or food additives. Despite these advantages, it still remains that these are complex of proteins that may act as strong antigens and are unlikely to be immunologically inert. Although their antibacterial activity is a significant achievement, establishing their biosafety is crucial to confirm their potential utility in both clinical and industrial settings. Unlike bacteriophages, tailocins cannot amplify at the site of infection and therefore lack the self-propagating properties that often contribute to the effectiveness of phage therapy. Consequently, higher doses or repeated administration may be required to achieve sustained antibacterial activity. These differences suggest that tailocins and bacteriophages should be viewed as complementary rather than competing antibacterial platforms, each offering distinct advantages depending on the therapeutic context.

RBPs can also be combined with other phage-derived proteins, such as endolysins, to achieve synergistic effects. Endolysins are primarily effective against Gram-positive bacteria due to their thick, readily accessible peptidoglycan layer. However, when fused with RBPs forming so-called Innolysins, they have demonstrated activity against Gram-negative pathogens, including *E. coli* and *P. aeruginosa*. As with tailocins, Innolysins may be easier to implement in practice because they are non-replicating protein complexes that do not transfer genetic material, but as well in that issue the biosafety confirmation is essential. Endolysins are enzymes that promote bacterial cell lysis. This process is associated with the release of huge amount of LPS as well as other bacterial cells components. These factors can stimulate the immune system, and in the severe cases, contribute to the development of septic shock.

The most innovative research appears to involve the use of advanced molecular models based on modeling approaches, which can undoubtedly lead to new discoveries. In particular, RBPseq platform may improve phage tail proteins annotation and deeper understanding of their mechanism of action. Furthermore, a comprehensive understanding of the structural relationships within this group of proteins may pave the way for advances in phage therapy itself. While *in silico* analysis play crucial role in modern structural biology, they cannot fully substitute for experimental validation. However, ongoing advances in these methodologies are likely to significantly accelerate structural studies and serves both as a starting point and complementary framework for experimental investigations of protein architecture.

A recent example of RBPs application involves their fusion with the Fc fragment of antibodies in efforts to develop novel immunotherapeutics. This approach stems from the functional similarity between bacteriophage RBPs and antibodies since—both are designed to recognize and bind specific targets. Although this strategy remains at an early stage of development, it appears highly promising. Therapeutic antibodies are widely used in the treatment of oncological and autoimmune diseases. However, relatively few are currently applied in the treatment of bacterial infections. The combination of RBPs with antibody fragments may therefore represent a promising new direction in antibacterial therapy.
